# An expert-based mapping of healthcare system strategies to support rational drug prescribing in primary care across 13 European countries

**DOI:** 10.1186/s12961-020-00605-w

**Published:** 2020-09-15

**Authors:** Alexandru M. Rotar, Michael J. van den Berg, Niek S. Klazinga

**Affiliations:** Department of Public Health, Academic Medical Center, Amsterdam Public Health Research Institute, University of Amsterdam, Meibergdreef 9, 1105 AZ Amsterdam, Netherlands

**Keywords:** Primary care, Prescription, Rational drug use

## Abstract

**Background:**

Irrational prescribing has received increasing attention among policy-makers to improve drug safety and effectiveness while avoiding economic waste. The policies intended to rationalise prescribing have been grouped by WHO under a taxonomy, classifying them into two types of strategies – (1) targeted approaches (micro level) and (2) system-oriented approaches (macro level). The extent to which countries implement strategies and the existing types is currently unknown. This paper explores the following research question via expert opinions: to what extent have European countries implemented strategies to support rational prescribing (targeted and system oriented) and what are the types implemented?

**Methods:**

We assessed the available information on policies intended to promote rational prescribing. We used the WHO taxonomy to explore our research question as the basis for a standardised questionnaire. The data were collected between August 2018 and April 2019. The questionnaire consisted of questions that solicited the opinion of experts on the implementation of prescribing control mechanisms in primary care in their respective countries. Experts were identified through the literature and relevant networks. The questionnaire was sent to 17 identified country experts from 17 different countries; 15 responded and 13 were used in our analysis. Answers were validated through follow-up correspondence, interviews and presentation at an OECD meeting.

**Results:**

Expert-reported data shows that all 13 countries included in our study have several mechanisms in place for enhancing rational prescribing in primary care. All approaches were reported to have been implemented in at least two countries. We identified two groups of countries, namely a small group of countries (*n* = 3) with fewer mechanisms in place and a larger group of countries (*n* = 10) with a large number of strategies with accompanying instruments at both the micro and macro levels.

**Conclusions:**

The data reported by the experts suggests that all 13 countries included in our study have several mechanisms in place for enhancing rational prescribing in primary care on both the micro and macro levels. With respect to the extent of mechanisms being in place, two groups of countries were identified. This initial mapping of strategies forms a basis for more in-depth research to be able to assess the impact of bundles of strategies on system and targeted level on rational drug prescribing in primary care in Europe.

## Background

In 1985, WHO defined the rational use of medicines as a process in which “*patients receive medications appropriate to their clinical needs, in doses that meet their own individual requirements, for an adequate period of time, and at the lowest cost to them and their community*” [1]. Later on, the World Bank proposed a new definition by adding the concepts of (1) drug use based on scientific data and (2) societal financial ability [2]. The irrational use of medicines is also acknowledged as a problem faced by many health systems across the world [3]. WHO estimates that 50% of all medicines across the world are inappropriately prescribed, dispensed or used [4], which has fuelled global recognition of both the underuse and overuse of medicines leading to poor health outcomes [5, 6].

Over-prescription (overuse) often results from the system’s undesired effects of financial incentives towards prescribers. Underuse is generally caused by poor access, health system delivery problems, (e.g. logistics, financial constraints, physicians’ awareness and skill level), or by patients not accessing, postponing or declining treatment [6]. The supply side (prescribers) is the main source of overuse, while patients (demand side) can drive both underuse and overuse [7]. The unintended effects have consequences on the population’s well-being by causing disabilities and loss of life-years but also on the financial resources use of patients and governments [5, 6].

In this context, the rational use of medicines plays an important role in today’s international policy debate. The global threat of antimicrobial therapy overuse resulting in resistance to antibiotics is one major policy challenge [8–10]. Furthermore, the debate on overuse has been expanded more recently towards benzodiazepines and opioids [11–13]. Consequently, irrational prescribing has been brought to the attention of policy-makers to improve the safety and effectiveness of drugs while avoiding economic waste [14–16]. Reported medical goods spending in European Union countries vary between 10% and 44% of the total national spending on healthcare [4, 17–19].

In an attempt to limit prescribing and tackle financial waste, different initiatives have been taken. At hospital level, clinical governance models were implemented to support evidence-based protocols and evaluation of compliance while, in making decisions on the added value of pharmaceuticals, health technology assessment has become a common approach [20, 21]. However, the concerns related to safety, effectiveness and economic waste in primary care are strongly influenced by irrational use. Given the importance of the primary care setting as the first point of contact for patients and drug prescribing practices and being the setting in which most chronic patients receive continuous care, rational drug prescribing is as important as in the hospital setting. Irrational prescribing, as the antimicrobial therapy overuse, is perceived to be caused by loosely regulated prescription requirements (e.g. protocols, audit-feedback) in primary care associated with less rational behavioural patterns and levels of knowledge on appropriate use (e.g. professional training and culture, personal experience, knowledge about availabilities and patients’ health literacy) [7, 18]. In the efforts to rationalise prescribing, as a large portion of total prescribing occurs in the community, outside of hospitals, primary care plays an important role [14, 22]. In the United Kingdom alone, despite a decreasing trend in the both share and value, National Health System data show that, in 2017, primary care accounts for more than 50% of the total proportion of estimated costs at list price [23].

Saini et al. [24] distinguished three main drivers of under- and overuse, namely (1) money and finance, (2) knowledge bias and uncertainty, and (3) power and human relationships, all acting at a global, national, regional and local/individual level. In attempts to improve prescribing, WHO classified the existing policies aimed to limit inappropriate prescribing and enhance the rational use of medicines in their publication Managing Access to Medicines and other Health Technologies [25]. The conceptual framework from WHO classifies policy measures as two types of strategies, namely as (1) targeted approaches (micro level) and (2) system-oriented approaches (macro level). Targeted approaches are strategies with a clinical focus (continuous medical education and continuous profession development) and at the health service level (managerial interventions such as limited procurement lists, cost information, therapy packaging). The clinical profile approaches seek to inform prescribers, while the health service-oriented approaches aim at regulating practice. At a system level, rational prescribing is enhanced by economic and regulatory interventions [25].

Knowing which combination of strategies (bundles) countries take in tackling inappropriate use of medicines can help to identify opportunities for international learning. Currently, there is no overview of the extent to which countries implement different bundles of strategies and the types they have in place on both system and targeted level. Consequently, this paper aims to obtain an initial answer on the following research question – to what extent have European countries implemented strategies to support rational prescribing (targeted and system-oriented) and what are the types implemented?

## Methods

We have made an assessment of the available information on policies that intend to promote rational prescribing. For our research, we adapted the WHO taxonomy to explore the research question by applying it as the basis for a standardised descriptive close-ended questionnaire (see Supplementary file 1) [25]. Subsequently, the questionnaire was pre-tested and sent to experts from 17 countries (15/17 response rate). The experts were identified and recruited from the Drug Utilization Review network^1^ and participants of a WHO Observatory Summer School on Quality of Care.^2^ We also used our personal research and policy networks to identify additional experts to expand the number of European countries and knowledge sources per country. The data collection process started in August 2018 and was completed in April 2019. The questionnaire consisted of questions that solicited the experts’ opinion on the implementation of prescribing control mechanisms in primary care in their respective countries. The key experts identified were selected based on their experience and their affiliation to organisations that are important and knowledgeable players in this field (e.g. academic institutions, ministries of health, practitioner organisations, etc.). During the data collection process, some of the contacted experts also engaged additional experts from their country, chosen based on their level of expertise and knowledge in the field, to assist in providing the required information. For validation purposes, once a questionnaire was received, we verified the answers with what was known from public documents and had active interaction with the responders via email and follow-up phone interviews. More in-depth discussions took place, especially where experts were unsure about the answers. Out of the 15 responders, we excluded 2, since these experts did not consider themselves knowledgeable enough to provide sufficient answers on both the system and targeted level and alternative experts for their country could not be identified or were non-responsive. Finally, the findings received per country were presented to the OECD Health Care Quality and Outcomes expert group in June 2019, in Paris, to provide feedback on the findings^3^ and hence serve as an additional means of validation.

## Results

Table 1 summarises the data reported by experts. It shows that (1) all countries included in our study have several mechanisms in place for enhancing rational prescribing in primary care and (2) all approaches are implemented in at least two countries in our group. Table 1 also shows the extent to which countries report to have implemented targeted and system-oriented strategies. Two groups were identified – a small group of countries with fewer mechanisms in place and a large group of countries that have a large number of strategies in place with accompanying instruments at both the micro and macro levels.

**Table 1:**
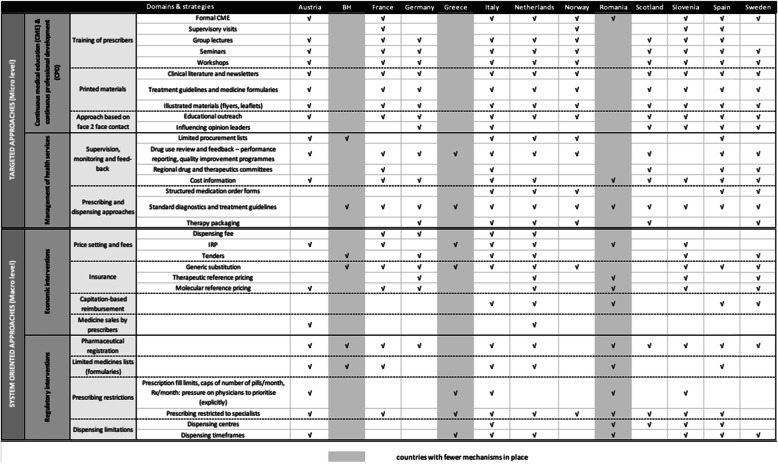
Overview of strategies to enhance rational prescribing in primary care in 13 countries

The small group of countries consists of Bosnia and Herzegovina, Greece and Romania, out of which Romania appears to have in place the highest number of mechanisms, mainly at a macro level (system approaches). The only common element among these three countries is that they have in place standard diagnosis and treatment guidelines (targeted approach). The macro level approaches reported to exist in Bosnia and Herzegovina are also reported to be in place by Greece (generic substitution) and Romania (pharmaceutical registration and limited medicines lists), except for the mechanism of tenders. The most common elements shared among Greece and Romania are international reference pricing, prescription fill limits, caps of number of pills per month, prescriptions per month, prescribing restricted to specialists and dispensing timeframes.

The larger group of countries consists of the remaining 10 from our list, namely Austria, France, Germany, Italy, the Netherlands, Norway, Scotland, Slovenia, Spain and Sweden. Within this second group, we distinguish three sub-groups, as follows: (1) Norway and Scotland appear to have almost all studied strategies on the micro level in place (targeted approaches), with fewer complementing macro level strategies; (2) Austria, France, Germany, Spain and Sweden have a wide range of both micro and macro level strategies; and (3) Italy, Netherlands and Slovenia, for which the country experts reported the existence of almost all types of strategies.

At the micro level, all 10 countries have implemented seminars, workshops, clinical literature and newsletters, treatment guidelines and medicine formularies, illustrated materials and educational outreach while, at a macro level, none of the studied mechanisms are implemented in all countries. All the approaches are implemented in at least half of the countries in this group (*n* = 5). One exception is supervisory visits, implemented in France, Norway, Slovenia and Spain. The second exemption, which is an economic disincentive for rational prescribing, medicines sales by prescribers, is reported to be implemented in Austria and the Netherlands in remote or rural areas where there are no pharmacies.

## Discussion

This study aims to provide an initial expert-based mapping of the extent to which European countries have implemented targeted and system-oriented rational prescribing strategies in their primary care systems and what bundles and types of strategies have been implemented. To our knowledge, such a system-based approach has not been attempted before. According to the country experts who responded to the survey, our study shows two main findings. Firstly, all countries have, according to the reporting experts, several mechanisms in place to enhance rational prescribing in primary care. Secondly, all the identified approaches previously mentioned are implemented in at least one of the studied countries.

For this study, we performed a literature and general policy documents search. We identified a taxonomy in a WHO publication, on which we built the questionnaire for our study. Although there is a fair amount of literature on the effectiveness of specific strategies, scientific evidence on the impact of simultaneously applying different bundles of strategies at the micro and macro levels was not identified. The results of this study are based on an expert opinion questionnaire with follow-up email or phone contact. We have chosen this approach to provide a broad general insight that can serve as the basis for more detailed studies such as more substantive consultation of groups of stakeholders/experts per country, in depth-interviews and more in-depth qualitative and quantitative exploration of the nature, scope and impact of the various bundles of strategies in their contexts. Future international comparative studies could focus on the relation over time between existing bundles of strategies on both micro and macro level as related to the actual reported prescribing rates for primary healthcare. Our findings are subject to the level of knowledge, oversight and opinions of the responding expert in each country and may not reflect a fully accurate picture of their country. However, it sufficed for the aim of mapping strategies and, in our overall conclusions, we have taken potential limitations of this expert opinion-based approach into account. Like in any expert opinion-based study, such results are based on personal judgements without any control on confounding factors [26]. We tried to mitigate a possible bias by asking about the concrete nature and existence of strategies through additional questions in a follow-up correspondence and interviews. Our study is an international comparative study. Consequently, the reliability and validity of constructs may also be hampered by health systems specificities that were not all explored in-depth.

Despite these limitations, this study is unique by way of offering a first attempt at mapping the use of existing bundles of prescribing strategies in primary care across countries and offers an overview allowing for further cross-country comparison. This study sets the basis for more in-depth quantitative and qualitative research on rational prescribing in primary care and may help policy-makers to understand the large differences between countries in prescribing rates as reported by international studies and organisations [18, 27]. For countries that want to improve their strategies for rationalising prescribing in primary care, this study can be a reference to further implement rational prescribing mechanisms.

There is a need for further research. This initial study is based on expert opinion, which is important to be validated further since opinions may differ (e.g. by literature review and Delphi panels). An example is the existence of papers on the implementation of formularies in Sweden and Scotland whilst the experts judged and reported that these were not available [28, 29]. However, this fact does not change the overall conclusions and, by publishing our overview scheme in the public domain, further validation and completion is possible.

Additionally, the sample of countries included in this study presents opportunities for expanding to a broader international group. Our international comparative research can help understand some of the existing gaps in rational prescribing strategies in primary care settings across countries and help fill them, but it also should be accompanied by quantifications of the effects of the implemented approaches. The continuous monitoring of the further development and implementation of bundles of strategies at the micro and macro levels is needed to help understand the reasons behind the existing (international) major differences in data on prescribing volumes such as the OECD Health at a Glance publication [18].

## Conclusions

The data reported by the experts shows that all countries included in our study have several mechanisms in place, at both the micro (targeted approaches) and macro (system approaches) levels, to enhance rational prescribing in primary care and that all approaches are implemented in at least two countries in our group. We have also identified in our sample two groups of countries – a small group of countries with fewer mechanisms in place and a large group of countries that have a large number of strategies (at both the micro and macro levels) in place. Our study shows that strategies for enhancing rational prescribing do not exist in isolation at one level of the system. To address the differences in prescribing rates between countries, it is necessary to learn more about the relative effectiveness of bundles of strategies to rationalise the prescribing practices that countries have been implementing. Macro level policies should be considered in combination with the operational tools at clinical and practice level as done in this study. This initial mapping of strategies forms a basis for further qualitative and quantitative research to be able to assess the impact of bundles of strategies at the system and targeted level on rational drug prescribing in primary care in Europe.

## Supplementary information

**Additional file 1: Supplementary file 1.**

## Data Availability

All data generated or analysed during this study are included in this published article.

## Footnotes

1 This is a network of researchers working on prescribing.

2 The European Observatory on Health Systems and Policies organises the annual Venice Summer School for policy-makers from different countries. In 2018, the Observatory Venice Summer School had quality of care as its main topic.

3 The OECD has a working party on Health Care Quality and Outcomes that discussed several papers on prescribing in June 2018 in Paris, France.
