# Supplementary material for: An expert-based mapping of healthcare system strategies to support rational drug prescribing in primary care across 13 European countries
Source: Health Res Policy Syst. 2020 Sep 15;18:102. doi: 10.1186/s12961-020-00605-w (PMC7493959; doi:10.1186/s12961-020-00605-w)
Supplement: Supplementary file 1 — Additional file 1: Supplementary file 1. [file 12961_2020_605_MOESM1_ESM.docx]

| **Study on national strategies to enhance rational prescribing of medication in primary care** | | | | |
| --- | --- | --- | --- | --- |
|  |  | |  | |
| **Country:** | |  | |  |
| Please provide us with your contact information: | | | |  |
| **Name:** | |  | |  |
| **Title:** | |  | |  |
| **Organization:** | |  | |  |
| **Postal address:** | |  | |  |
| **Email:** | |  | |  |
| **Telephone:** | |  | |  |
| **Function:** | |  | |  |
| **Thank you for agreeing to complete this questionnaire, which seeks to describe and assess the existing prescribing control mechanisms in primary care** | | | | |

1. **TARGETED APPROACHES**
2. **Is rational prescribing part of continuous educational programmes (CME, CPD) of physicians working in primary care?** (Y/N)

**If YES**, please tick any of the following strategies applied on a regular basis in your country:

| **1.1. Training of prescribers** | **Y/N** | **Other relevant information** |
| --- | --- | --- |
| Formal CME |  |  |
| Supervisory visits |  |  |
| Group lectures |  |  |
| Seminars |  |  |
| Workshops |  |  |
| **1.2. Printed materials** |  |  |
| Clinical literature and newsletters |  |  |
| Treatment guidelines and medicine formularies |  |  |
| Illustrated materials (flyers, leaflets) |  |  |
| **1.3. Approach based on face to face contact** |  |  |
| Educational outreach |  |  |
| Influencing opinion leaders |  |  |

1. **Is rational prescribing part of the management of the health service in primary care? (Y/N)**

**If YES**, please tick any of the following implemented strategies:

| **2.1. Supervision, monitoring and feedback** | **Y/N** | **Other relevant information** |
| --- | --- | --- |
| Limited procurement lists |  |  |
| Drug use review and feedback – performance reporting, quality improvement programmes |  |  |
| Regional drug and therapeutics committees |  |  |
| Cost information |  |  |
| **2.2. Prescribing and dispensing approaches** |  |  |
| Structured medication order forms |  |  |
| Standard diagnostics and treatment guidelines |  |  |
| Therapy packaging |  |  |

**Other relevant information (please also use if necessary the back of the paper):** ……………………...………………………………………………………………………………………………………………………….…………………………………………………………………………………………………………………………………………………..………………………………………………………………………………………………………………………………………………………………………………………………………………………………………………………………………………………………...

1. **SYSTEM ORIENTED APPROACHES**
2. **Is rational prescribing addressed in primary care through economic interventions? (Y/N)**

**If YES**, please tick any of the following implemented strategies:

| **3.1. Price setting and fees** | | | **Y/N** | **Other relevant information** |
| --- | --- | --- | --- | --- |
| Dispensing fee | | |  |  |
| International reference pricing | | |  |  |
| Tenders | | |  |  |
| **3.2. Insurance** | | |  |  |
| Generic substitution | | |  |  |
| Therapeutic reference pricing | | |  |  |
| Molecular reference pricing | | |  |  |
| **3.3. Capitation-based reimbursement** | | |  |  |
|  |  |  |  |  |
| **3.4. Medicine sales by prescribers** | | |  |  |
|  |  |  |  |  |

1. **Is rational prescribing addressed in primary care thorough regulatory interventions? (Y/N)**

**If YES**, please tick any of the following implemented strategies:

| **4.1. Pharmaceutical registration** | | | **Y/N** | **Other relevant information** |
| --- | --- | --- | --- | --- |
|  |  |  |  |  |
| **4.2. Limited medicines lists (formularies)** | | |  |  |
|  |  |  |  |  |
| **4.3. Prescribing restrictions** | | |  |  |
| Prescription fill limits, caps of number of pills/month, Rx/month: pressure on physicians to prioritise (explicitly) | | |  |  |
| Prescribing restricted to specialists | | |  |  |
| **4.4. Dispensing limitations** | | |  |  |
| Dispensing centres | | |  |  |
| Dispensing timeframes | | |  |  |

**Other relevant information (please also use if necessary the back of the paper too):** ……………………...……………………………………………………………………………………………………………………………….……………………………………………………………………………………………………………………………………………………………………………………………………………………………………………………………………………………………….………………………………………………………………………………………………………………………………………………….………………………………………………………………………………………………………………………………………………….………………………………………………………………………………………………………………………………………………….………………………………………………………………………………………………………………………………………………….………………………………………………………………………………………………………………………………………………….………………………………………………………………………………………………………………………………………………….………………………………………………………………………………………………………………………………………………….………………………………………………………………………………………………………………………………………………….………………………………………………………………………………………………………………………………………………….………………………………………………………………………………………………………………………………………………

1. **Are you aware of any other prescribing control mechanisms implemented at GPs level in your country? (Y/N)**

**If YES please mention them below:**

………………………..

………………………..

………………………..

1. **Are you aware of any other prescribing control mechanisms aimed at patient education implemented in your country? (Y/N)**

**If YES please mention them below:**

………………………..

………………………..

………………………..

1. **Is there any pharmaceutical consumption data for primary care available in your country? (Y/N)**

**If YES, please indicate the source:**

………………………..

………………………..

………………………..
